# Gastroparesis might not be uncommon in patients with diabetes mellitus in a real-world clinical setting: a cohort study

**DOI:** 10.1186/s12876-023-03106-6

**Published:** 2024-01-11

**Authors:** Jeongmin Lee, Hye Lim Park, Su Young Park, Chul-Hyun Lim, Min-Hee Kim, Jung Min Lee, Sang-Ah Chang, Jung-Hwan Oh

**Affiliations:** 1 Division of Endocrinology and Metabolism, Department of Internal Medicine, Eunpyeong St. Mary’s Hospital, College of Medicine, The Catholic University of Korea, Seoul, 03312 South Korea; 2 Division of Nuclear medicine, Department of Radiology, Eunpyeong St. Mary’s Hospital, College of Medicine, The Catholic University of Korea, Seoul, 03312 South Korea; 3 Division of Gastroenterology, Department of Internal Medicine, The Catholic University of Korea, Seoul, 03312 Republic of Korea

**Keywords:** Diabetes mellitus, Diabetic neuropathies, Gastroparesis, Gastric emptying, Radionuclide imaging

## Abstract

**Background:**

This study investigated the frequency of diabetic gastroparesis and associated risk factors in a real-world clinical setting.

**Methods:**

This retrospective cross-sectional study included patients who underwent assessments of solid gastric emptying time (GET) by technetium-99 m scintigraphy between May 2019 and December 2020. We categorized patients into three groups according to gastric retention of technetium-99 m: rapid (< 65% at 1 h or < 20% at 2 h), normal (≤60% at 2 h and/or ≤ 10% at 4 h), and delayed (> 60% at 2 h and/or > 10% at 4 h).

**Results:**

Patients with diabetes mellitus (DM) were more likely to show abnormal GET than those without DM (119 [70.8%] vs. 16 [44.4%]). The mean glycated A1c was 10.3% in DM patients. DM patients with normal GET were significantly younger (57.2 years, *P* = 0.044) than those with delayed (65.0 years) or rapid GET (60.2 years). Fasting glucose levels were the lowest in the normal GET group and the highest in the rapid GET group (delayed: 176.3 mg/dL, normal: 151.2 mg/dL, rapid: 181.0 mg/dL, *P* = 0.030). However, glycated A1c was not significantly different among the delayed, normal, and rapid GET groups in patients with DM. Patients with delayed and rapid GET showed a higher frequency of retinopathy (6.0 vs. 15.5%, *P* = 0.001) and peripheral neuropathy (11.3 vs. 24.4%, *P* = 0.001) than those with normal GET. In the multinomial logistic regression analysis, retinopathy demonstrated a positive association with delayed GET, while nephropathy showed a significant negative correlation.

**Conclusion:**

DM gastroparesis in the clinical setting was not uncommon. Abnormal GET, including delayed and rapid GET, was associated with DM retinopathy or peripheral neuropathy.

**Supplementary Information:**

The online version contains supplementary material available at 10.1186/s12876-023-03106-6.

## Background

Diabetes mellitus (DM) is becoming increasingly prevalent in Korean adults, affecting up to 6.05 million individuals (16.7%) according to the 2021 Diabetes Fact Sheet in Korea [1]. As complications arising from DM, such as macrovascular and microvascular issues (e.g., neuropathy), become more prevalent, preventing these complications has become a significant concern. Among the various types of diabetic neuropathy, diabetic gastroparesis (DGP) was documented first by Rundles in 1945 [2] and Kassandra coined the term “*gastroparesis diabeticorum*” [3]. Gastroparesis is a chronic symptomatic gastric disorder characterized by impaired gastric motility in the absence of outlet obstruction [4, 5] and DM is one of the leading causes of gastroparesis [5, 6]. The manifestations of DGP include early satiety, postprandial fullness, nausea, vomiting, and abdominal pain [7]. The pathophysiology of DGP is postulated to involve hyperglycemia or extreme hypoglycemia-induced impairment of gastrointestinal (GI) vagal dysfunction from the loss of Cajal’s interstitial cells and enteric glial cells. The regeneration of signaling is influenced by oxidative stress and advanced glycation end products, as well as neuroimmune mechanisms [8]. The diagnosis of DGP is based on normal esophagogastroduodenoscopy findings and an abnormal gastric emptying time (GET) [4]. However, patients with DM who have related symptoms may show normal or rapid GET without symptoms, making the association between GI manifestations and GET ambiguous.

Although DGP is a well-known neuropathic complication of DM, the prevalence of DGP remains unclear due to the requirement for specialized laboratories for scintigraphy. Moreover, DGP is often under-recognized and poorly managed. Currently, studies on DGP prevalence in patients with DM are limited. According to a recent Asian study, the diagnosis of gastroparesis continues to be a challenge [9]. In this study, we aimed to investigate the frequency of DGP and the risk factors of DGP in DM patients through a retrospective study using real-world evidence.

## Methods

### Study design and selection of participants

This retrospective analysis of electronic medical records (EMRs) focused on patients admitted for glycemic control at the Department of Endocrinology and Metabolism of Eunpyeong St. Mary’s Hospital between May 2019 and December 2020. To define diabetes, the International Classification of Diseases, 10th Revision (ICD-10) was used, with the following codes: E10 (type 1 diabetes mellitus) and E11-E14 (type 2 diabetes mellitus). The study included hospitalized patients who underwent an assessment of solid gastric emptying times (GET) by technetium-99 m scintigraphy, with exclusion criteria consisting of 1) a swallowing disorder; 2) a malignant tumor in the GI or hepatobiliary tract or pancreas; 3) pregnancy or breastfeeding; 4) a previous GI tract operation except for simple perforation, appendectomy, cholecystectomy, hysterectomy, benign tumor resection using endoscopy, and endoscopic polypectomy; 5) a gastric electrical stimulator device; 6) severe liver disease or chronic renal disease; and 7) a history of alcohol or drug abuse.

During hospitalization for glycemic control, patients were treated with insulin combination therapy. For a minimum of 3–4 days during the hospitalization period, none of the patients received oral hypoglycemic agents or Glucagon-like peptide-1 agonists.

### Measurements and definitions

Data on basic characteristics at the time of admission, including age, sex, height, weight, and body mass index (BMI), were extracted. The BMI was calculated by dividing the weight in kilograms by the square of the height in meters (kg/m^2^). Laboratory tests included glycated A1c (HbA1c), serum fasting glucose (FG), postprandial glucose (PPG), blood urea nitrogen, serum creatinine (Cr), aspartate aminotransferase (AST), and alanine aminotransferase (ALT). All measurements were performed using an automated blood chemistry analyzer (Hitachi 747; Hitachi, Tokyo, Japan). HbA1c was measured by high-performance liquid chromatography using Diabetes Control and Complications Trial-aligned methods (Tosoh-G8; Tosoh, Tokyo, Japan).

### Direct chart review and data quality management

To ensure the accuracy of the data, a direct chart review was conducted to confirm whether the patients were admitted for glycemic control. One researcher conducted the chart review and modified the data as necessary. The modified data were kept separately and processed using the same protocol.

### Privacy protection

None of the data included personally identifiable information, including the patient’s name and social security number. Instead, a responsible investigator assigned a temporary number to the patient’s hospital registration number. After the analysis, the registration number was removed. Only the responsible investigator had access to the file linking the hospital registration number with the temporary number. If a chart review was necessary after statistical processing, the responsible investigator could access the hospital information. All data were stored in encrypted files on a secure computer that was only accessible to the investigator. Since this study only utilized the EMRs of patients who had completed treatment, there was no risk to the patients’ physical or mental well-being.

### Tc-99 m phytate GET scan protocol and imaging analysis

All patients fasted for at least 12 hours prior to the Tc-99 m phytate GET scan. Patients were requested to discontinue taking drugs affecting the emptying time such as prokinetic agents, opiates, antispasmodic agents and benzodiazepines 48 hours prior to the scan.

A meal was served, consisting of a radiolabeled scrambled egg (one whole egg with 37 MBq of Tc-99 m phytate), 2 pieces of *gimbap*, and 300 mL of water. The meal had to be consumed within 10 minutes. Anterior and posterior planar images were obtained immediately after completion of eating. Scans were acquired in a 128 × 128 matrix using a dual-head gamma camera with a low-energy high-resolution (LEHR) collimator (Symbia Evo, Siemens Medical Solutions, Knoxville, TN, USA). All patients were in an upright position during the scan and were scanned for 2 minutes per frame at 0, 20, 40, 60, 80, and 120 minutes after the meal. Delayed 4-hour images were acquired when gastric retention at the 2-hour scan exceeded 40%.

Region of interest (ROI) was manually drawn including the stomach on the anterior and posterior images. The square root of the count in the anterior and posterior ROIs (geometric mean) was used to calculate gastric empting. We categorized patients into three groups according to gastric retention of technetium-99 m: rapid (< 65% at 1 h or < 20% at 2 h), normal (≤60% at 2 h and/or ≤ 10% at 4 h), and delayed (> 60% at 2 h and/or > 10% at 4 h) (Supplemental Fig. 1).

### Statistical analyses

The baseline characteristics are presented as the mean with standard deviation for continuous variables for which a normal data distribution was confirmed, and as numbers and percentages for categorical variables. Characteristics were compared between patients with and without DM using the independent t-test for continuous variables and the chi-square test for dichotomous variables. Clinical characteristics were compared between the groups stratified by GET using analysis of variance for continuous variables and the chi-square test for categorical variables. One-versus-rest logistic regression was used to assess the association between GET and the variables. We conducted a multinomial logistic regression analysis to assess the association between GET and Type 2 DM, adjusting for duration of DM, sex, age, BMI, HbA1c, FG, PPG, glucose before GET, and GFR (estimated glomerular filtration rate). The results include beta coefficients (β), 95% confidence intervals (CI), and *P* values for each covariate. In the subanalysis, a sex-stratified investigation was conducted using multinomial logistic regression. A *P* value of < 0.05 was considered statistically significant. All statistical analyses were performed using SAS version 9.4 (SAS Institute, Cary, NC, USA).

## Results

### Baseline characteristics of study subjects

After exclusion, a total 168 patients with DM and 36 subjects without DM were finally included in this analysis. The characteristics of the study subjects are summarized in Table 1. Female show more frequent distribution in our study than male. The mean age were 62.1 years in patients with DM and 51.5 years in subjects without DM, respectively.

**Table 1:** Baseline clinical characteristics of patients with diabetes and without diabetes

Characteristics	DM patients (*n* = 168)	Subjects without diabetes (*n* = 36)	*P*-value
Sex, male, n (%)	72 (42.9)	12 (33.3)	0.386
Age, years	62.1 ± 15.3	51.5 ± 16.0	< 0.001
BMI, kg/m^2^	25.8 ± 4.4	23.0 ± 3.8	< 0.001
Glycated A1c, %	10.3 ± 2.3	5.8 ± 1.1	< 0.001
Fasting glucose, mg/dl	179.6 ± 72.1	110.9 ± 18.7	< 0.001
Postprandial glucose, mg/dl	276.0 ± 90.8	191.0 ± 63.6	0.304
Serum glucose before GET, mg/dl	135.5 ± 30.8	106.7 ± 10.5	< 0.001
AST,U/L	27.7 ± 17.7	21.8 ± 5.9	0.001
ALT, U/L	30.2 ± 23.5	18.4 ± 8.0	< 0.001
BUN, mg/dl	19.8 ± 12.7	14.4 ± 4.6	< 0.001
Serum Cr, mg/dl	1.0 ± 1.1	0.8 ± 0.2	0.012
GFR, ml/min/1.73m^2^	92.5 ± 78.2	92.6 ± 18.1	0.994
Gastric emptying time			0.011
Normal, n (%)	49 (29.2)	20 (55.6)	
Delayed, n (%)	39 (23.2)	4 (11.1)	
Rapid, n (%)	80 (47.6)	12 (33.3)	

Note: Data are presented as means or as distribution inclusion percentages

*ALT* alanine aminotransferase: *AST* aspartate aminotransferase: *BMI* body mass index: *BUN* blood urea nitrogen: *Cr* creatinine: *DM* diabetes mellitus: *GET* gastric emptying time: *GFR* estimated glomerular filtration

GET categorization using gastric retention of technetium-99 m: rapid (< 65% at 1 hr. or < 20% at 2 hrs), normal (6 ≤ 0% at 2 hrs and/or ≤ 10% at 4 hrs), delayed (> 60% at 2 hrs and/or > 10% at 4 hrs)

Among 168 of DM patients, 166 (98.9%) were categorized into type 2 DM (data not shown) and mean HbA1c was 10.3%. BMI, FG, glucose before GET, AST, and ALT was higher in DM patients than in non-DM patients. PPG and GFR did not differ between DM and non-DM patients. Abnormal finding in GET including rapid and delayed passage were more prevalent in DM patients with significance [119 (70.8%) vs 16 (44.4%), *P* = 0.011].

### Clinical characteristics according to GET in DM patients

**Fig. 1:**
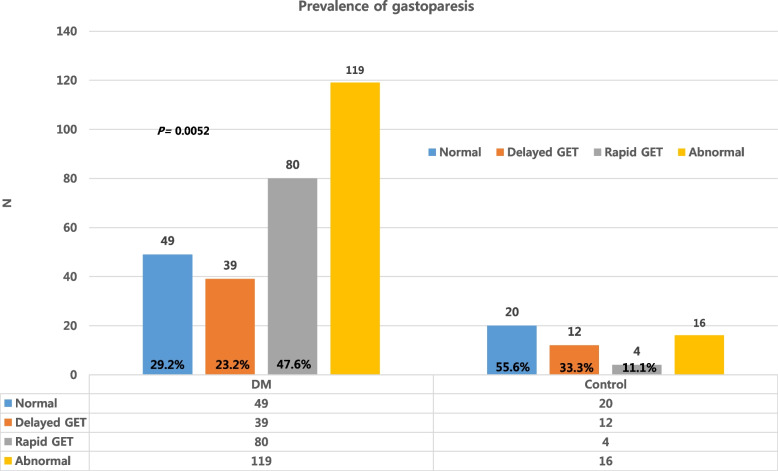
frequency of gastroparesis. 119 patents (70.8%) showed abnormal results of GET with DM showed abnormal GET findings in this study

The majority of patients with DM (98.8%) in this study had type 2 DM, and 119 patients with DM (70.8%) showed abnormal GET results (delayed GET: 23.2% vs. rapid GET: 47.6%) (Fig. 1). Abnormalities in GET were more common in older patients than younger patients, but there was no significant difference in the duration of DM among those with delayed GET, normal GET, and rapid GET. FG levels were significantly lower in patients with normal GET (151.2 mg/dL) than in the abnormal GET groups (176.3 mg/dL for delayed GET and 181.0 mg/dL for rapid GET, *P* = 0.030). However, HbA1c, PPG, and glucose levels before GET were similar among the different groups. The most prevalent DM complications were retinopathy and peripheral neuropathy, with the rapid GET group showing higher frequency than the delayed GET group (Table 2).

**Table 2:** Comparison of clinical characteristics according to gastric emptying time

Characteristics	Gastric emptying time			*P*-value
	Delayed (*n* = 39)	Normal (*n* = 49)	Rapid (*n* = 80)	
DM type				0.057
Type 1 DM, n (%)	0	0	2 (100)	
Type 2 DM, n (%)	39 (23.5)	49 (29.5)	78 (47.0)	
Duration of DM (years)	9.4 ± 9.8	7.6 ± 10.3	9. 0 ± 10.8	0.591
Age	65.0 ± 13.6	57.2 ± 18.1	60.2 ± 14.7	0.044
BMI, kg/m^2^	24.9 ± 3.4	25.1 ± 4.8	25.7 ± 4.5	0.566
Glycated A1c, %	10.3 ± 2.7	9.6 ± 2.4	10.2 ± 2.5	0.285
Fasting glucose, mg/dl	176.3 ± 87.5	151.2 ± 59.4	181.0 ± 68.7	0.030
Postprandial glucose, mg/dl	271.5 ± 89.4	262.8 ± 81.1	284.3 ± 97.1	0.479
Serum glucose before GET, mg/dl	133.6 ± 28.6	132.9 ± 33.4	132.0 ± 29.9	0.964
GFR, ml/min/1.73m^2^	1.3 ± 2.0	0.9 ± 0.4	1.0 ± 0.6	0.106
DM complication, n(%)				
Macrovascular	14 (8.3)	20 (11.9)	30 (17.9)	0.871
Retinopathy	10 (6.0)	4 (2.4)	26 (15.5)	0.001
Nephropathy	2 (1.2)	4 (2.4)	8 (4.8)	0.627
Peripheral neuropathy	19 (11.3)	12 (7.1)	41 (24.4)	0.001
Autonomic neuropathy	20 (11.9)	17 (10.1)	39 (23.2)	0.026
Hypoglycemia	0 (0.0)	1(5.6)	1 (5.6)	N/A

Note: Data are presented as means or as distribution inclusion percentages

*ALT* alanine aminotransferase: *AST* aspartate aminotransferase: *BMI* body mass index: *DM* diabetes mellitus: *GET* gastric emptying time: *GFR* estimated glomerular filtration rate

* categorization using gastric retention of technetium-99 m: rapid (< 65% at 1 hr. or < 20% at 2 hrs), normal (6 ≤ 0% at 2 hrs and/or ≤ 10% at 4 hrs), delayed (> 60% at 2 hrs and/or > 10% at 4 hrs)

### An association between gastric emptying time and diabetes mellitus with associated covariate

In the linear regression with multinomial analysis, delayed GET positively correlated with DM retinopathy and hypoglycemia (β coefficient = 1.985, *P* = 0.007 and β coefficient = 19.752, *P* = 0.000, respectively), while DM nephropathy demonstrated a significantly negative correlation (β coefficient = − 2.924, *P* = 0.041) (Table 3). In rapid GET, there was positive correlation between GET and DM retinopathy and peripheral neuropathy, hypoglycemia (β coefficient = 1.994, *P* = 0.003 in DM retinopathy, β coefficient = 1.114, *P* = 0.021 in peripheral neuropathy, and β coefficient = 19.656, *P* = 0.000) However, there was no significant correlation between GET and duration of DM, sex, age, BMI, FG, PPG, GFR, and DM nephropathy.

**Table 3:** Multinomial logistic regression analysis of gastric emptying times considering diabetes mellitus and associated covariates

	Delayed GET β (95% CI)	*P*-value	Rapid GET β (95% CI)	*P*-value
Duration of DM	0.001 (− 0.06,0.06)	0.963	0.015 (− 0.04.0.06)	0.559
Sex, female	0.189 (−0.88,1.25)	0.727	− 0258 (−1.18.0.67)	0.584
Age	0.022 (−0.01,0.06)	0.258	−0.005 (− 0.04,0.03)	0.776
BMI	−0.100 (− 0.21,0.01)	0.079	− 0.051 (− 0.15,0.05)	0.302
Glycated A1c	0.128 (− 0.12,0.38)	0.318	0.168 (− 0.06,0.40)	0.147
Fasting glucose	0.006 (0.00,0.02)	0.264	0.005 (0.00,0.13)	0.253
Postprandial glucose	−0.002 (− 0.01,0.01)	0.599	− 0.001 (− 0.01,0.01)	0.793
Glucose before GET	− 0.005 (− 0.02,0.01)	0.564	− 0.006 (− 0.02.0.01)	0.371
GFR, ml/min/1.73m^2^	0.001 (− 0.01,0.01)	0.874	0.003 (− 0.01.0.02)	0.635
DM complication				
Macrovascular	−0.260 (−1.30,0.78)	0.629	−0.103 (− 1.03,0.82))	0.828
Retinopathy	1.985 (0.54,3.243)	0.007	1.994 (0.70,3.29)	0.003
Nephropathy	−2.924 (−5.72,-0.13)	0.041	−1.19 (−3.13,0.74)	0.227
Peripheral neuropathy	0.791 (−0.30,1.88)	0.153	1.114 (0.17,2.06)	0.021
Autonomic neuropathy	0.521 (−0.51,1.55)	0.323	0.778 (−0.14,1.70)	0.096
Hypoglycemia	19.752 (18.20, 21.31)	0.000	19.656 (18.10, 21.21)	0.000

*BMI* body mass index: *DM* diabetes mellitus: *GET* gastric emptying time: *GFR* estimated glomerular filtration rate

* categorization using gastric retention of technetium-99 m: rapid (< 65% at 1 hr. or < 20% at 2 hrs), normal (6 ≤ 0% at 2 hrs and/or ≤ 10% at 4 hrs), delayed (> 60% at 2 hrs and/or > 10% at 4 hrs)

### Subanalysis

We performed subgroup analyses according to sex (Table 4). In females, abnormal (delayed or rapid GET) GET did not show significant correlations with the duration of DM, age, BMI, HbA1c, FG, PPG, glucose before GET, or GFR. However, DM retinopathy and hypoglycemia exhibited a positive correlation with delayed GET (β coefficient = 2.884, *P* = 0.012 and β coefficient = 20.664, *P* = 0.000), while DM nephropathy demonstrated a significantly negative correlation (β coefficient = − 19.59, *P* = 0.000). In male participants, delayed GET and rapid GET was positively correlated FG (β coefficient = 0.029, *P* = 0.034). In the analysis of rapid GET, male participants exhibited positive correlations between rapid GET and DM retinopathy (β coefficient = 2.279, *P* = 0.042) and negative correlation with DM nephropathy (β coefficient = − 7.407, *P* = 0.000). In the male group, there were no patients with hypoglycemia, resulting in an estimated standard error of 0 and, consequently, an undefined *P* value.

**Table 4:** Multinomial logistic regression analysis of gastric emptying times considering diabetes mellitus and associated covariates according to sex

	Delayed GET β (95% CI)	*P*-value	Rapid GET β (95% CI)	*P*-value
**Female**				
Duration of DM	−0.020 (− 0.12,0.08)	0.691	0.029 (− 0.04.0.10)	0.404
Age	0.037 (−0.01,0.08)	0.134	0.037 (−0.01,0.09)	0.132
BMI	−0.013 (− 0.15,0.13)	0.860	0.00 (− 0.13,0.13)	0.998
Glycated A1c	0.191 (− 0.19,0.57)	0.322	0.073 (−0.29,0.43)	0.692
Fasting glucose	−0.001 (− 0.01,0.01)	0.930	0.002 (− 0.01,0.01)	0.706
Postprandial glucose	−0.002 (− 0.01.0.01)	0.643	0.001 (− 0.01,0.01)	0.738
Glucose before GET	−0.007 (− 0.03,0.02)	0.524	0.001 (00.02,0.02)	0.906
GFR, ml/min/1.73m^2^	0.008 (−0.02,0.03)	0.539	−0.314 (−2.13,1.50)	0.091
DM complication				
Macrovascular	−0.038 (−1.55,1.47)	0.961	0.063 (−1.25,1.38)	0.925
Retinopathy	2.884 (0.63,5.13)	0.012	1.491 (−0.53,6.51)	0.149
Nephropathy	−19.59 (− 19.59,-19.59)	0.000	−0.186 (−2.93,2.56)	0.894
Peripheral neuropathy	0.124 (−1.32,1.57)	0.866	0.943 (− 0.27,2.16)	0.127
Autonomic neuropathy	− 0.003 (−1.40,1.39)	0.997	0.306 (− 0.93,1.54)	0.628
Hypoglycemia	20.664 (19.11,22.22)	0.000	20.569 (19.01,22.13)	0.000
**Male**				
Duration of DM	−0.003 (−0.12,0.12)	0.964	0.021(−0.09,0.13)	0.708
Age	−0.012 (− 0.01,0.06)	0.741	− 0.058 (−1.12,0.00)	0.067
BMI	−0.370 (− 0.66,-0.08)	0.011	− 0.202 (− 0.44,0.03)	0.094
Glycated A1c	− 0.144 (− 0.64,0.35)	0.569	0.210 (− 0.26,0.68)	0.386
Fasting glucose	0.029 (0.00,0.01)	0.034	0.028 (0.00,0.05)	0.032
Postprandial glucose	−0.010 (− 0.03.0.01)	0.260	− 0.013 (− 0.03,0.00)	0.115
Glucose before GET	− 0.019 (− 0.05,0.01)	0.256	−0.021 (− 0.05,0.01)	0.167
GFR, ml/min/1.73m^2^	0.001 (−0.02,0.02)	0.939	0.003 (−0.02,0.03)	0.820
DM complication				
Macrovascular	0.897 (−1.09,2.88)	0.375	0.043 (−1.82,1.90)	0.964
Retinopathy	0.845 (−1.85,3.54)	0.539	2.279 (0.08,4.48)	0.042
Nephropathy	−2.456 (−5.34,0.43)	0.096	−7.407 (−10.22,-4.59)	0.000
Peripheral neuropathy	0.623 (−0.934,4.18)	0.213	1.977 (−0.38,4.33)	0.100
Autonomic neuropathy	−1.501 (−0.72,3.72)	0.185	1.952 (− 017,4.07)	0.071
Hypoglycemia	0.000	NA	0.000	NA

*BMI* body mass index: *DM* diabetes mellitus: *GET* gastric emptying time: *GFR* estimated glomerular filtration rate: *NA* not Available

* categorization using gastric retention of technetium-99 m: rapid (< 65% at 1 hr. or < 20% at 2 hrs), normal (6 ≤ 0% at 2 hrs and/or ≤ 10% at 4 hrs), delayed (> 60% at 2 hrs and/or > 10% at 4 hrs)

## Discussion

We demonstrated that 70.8% of patients with type 1 DM or type 2 DM had DGP, with a higher frequency observed in those with diabetic retinopathy or peripheral neuropathy. To our knowledge, this is the first study to investigate the frequency of DGP based on GET in a clinical setting in Korea. DGP is regarded as an autonomic neuropathy-related complication of DM, resulting in poor glycemic control and low health-related quality of life [10]. However, inconsistent findings have been reported regarding the epidemiology of gastroparesis due to the limited use of accurate diagnostic tools for GET. The differing rates may be related to the demographic or clinical parameters of the study population and diagnostic methods used. Recent guidelines also recommend scintigraphic gastric emptying as the standard test for evaluating gastroparesis in patients with upper GI symptoms [5].

Previous research using US population data reported DGP incidence rates of 5.2% in type 2 diabetes and 1.0% in type 1 diabetes, respectively [11, 12]. However, these rates might not reflect the incidence of DGP in other populations or clinical settings. Other studies have reported the prevalence rate of DGP to range from 25 to 65% [13–16]. These results, based on tertiary hospital settings, were similar to our findings, with 39% of DM patients showing delayed DGP. The definition of gastroparesis should include delayed gastric emptying. However, previous studies only focused on delayed GET and did not include rapid GET results. Recent concepts also call for attention to rapid gastric emptying which seems to be considered a complication of DM [17]. Rapid gastric emptying appeared in patients with dyspepsia, and it has been reported that diabetic patients were more likely to have rapid gastric emptying [18]. About one fifth of people with long-term diabetes experience rapid gastric emptying. The relationship between abnormal GET and dysglycemia in the context of DM is often bidirectional and complex. Chronic hyperglycemia is associated with increased superoxide dismutase levels, an enzyme that may elevate hydrogen peroxide production [19] and this oxidative stress impact the function of the nerves, hormones, interstitial cells of Cajal, and smooth muscles, potentially causing rapid gastric emptying [17]. Abnormal GET showed the impact on hyperglycemia. The rapid GET contributes to elevated FG and increased glycemic variability, encompassing PPG. Conversely, rapid GET may lead to a quicker influx of nutrients, including glucose, into the bloodstream. This accelerated nutrient absorption can contribute to high levels of PPG [20]. In previous study with 75 g oral glucose tolerance test, in individuals with impaired glucose tolerance and type 2 DM, GET is directly linked to glycemia at 30 and 60 minutes. Specifically, in type 2 DM there is a direct relationship at 120 minutes, a crucial time point for DM, where raid GET is associated with an increased glycemic response [21]. In contrast to the result according to frequency of abnormal GET, there was no significant correlation in FG and PPG in our study.

Our study found that the frequency of DGP in our cohort was higher than in the studies mentioned above. Surprisingly, we found that the rate of rapid GET was higher than that of delayed GET in DM patients (47.6% vs 23.2%). In this study, 56.3% of patients were women, which may have contributed to the higher frequency of DGP. This sex difference aligns with a recent analysis of a diabetic cohort with rapid GET [22]. The reasons for the higher incidence of DGP in women remain unclear, but estrogen regulation of the pathway for neuronal nitric oxide synthesis related to gastric motility may be a factor [23]. Hormonal fluctuations, particularly those related to the menstrual cycle, may influence gastric motility. Changes in estrogen and progesterone levels during the menstrual cycle could potentially affect the rate of GET [24]. During the luteal phase of the menstrual cycle, progesterone effects on the autonomic nervous system, and the net result is an increase in GET [25]. One study in postmenopausal women, increased level of electrical activity associated with gastric concentration rather than men [26]. However, the specific mechanisms underlying this heightened activity and its implications remain unclear and may require further investigation. Moreover, despite the higher frequency of gastric emptying abnormalities in women, the correlation between GET and DM did not show statistical significance according to sex in our study.

Another plausible factor contributing to the high rate of rapid GET is obesity. Increased pressure in the stomach and hormones such as insulin, ghrelin, and leptin affect gastric motility and induce rapid gastric emptying [27]. Our analysis is consistent with studies demonstrating that obesity, and particularly a high BMI, among patients with diabetes is a common characteristic of patients with rapid GET [28, 29].

One study showed that there was no correlation between symptoms and GET results in patients with functional dyspepsia and gastroparesis [30], so it is necessary to consider the clinical implications of rapid GET. In this study, the mean BMI in DM patients with rapid GET was 25.7 kg/m^2^, and the DM patients were, on average, almost obese according to the criteria used for Asian populations (24.9 kg/m^2^). The duration of DM and higher HbA1c were other factors associated with a higher frequency of DGP. As reported in previous research, hyperglycemia has an impact on GET even in the general population [31]. Hyperglycemia induces abnormal production of advanced glycation end products, which are the main cause of DM neuropathy [32]. Autonomic neuropathy has been implicated as the major mechanism underlying DGP [33]. Thus, autonomic dysfunction is correlated with GI vagal dysfunction. Our findings align with the proposal that autonomic dysfunction and peripheral neuropathy are associated with a higher frequency of DGP.

The main advantage of this EMR-based retrospective study is that it used real-world evidence based on data obtained from real clinical practice. Although our results generally agree with those of most previous studies, it is worth noting that rapid GET occurred more often in DM patients. However, several limitations should be acknowledged. First, this study was retrospective in nature and was based on EMR data. The resulting absence of information regarding patients’ symptoms reduces our confidence in the accuracy of correlations between symptoms and GET. To address this, future studies should use a valid questionnaire to assess GI symptoms accurately. Because this retrospective study was based on data from a single center, we could not confirm a causal relationship between DGP and various risk factors. Furthermore, our subjects were hospitalized DM patients, so it is unclear how prevalent DGP is in outpatient settings and whether the duration of DM and poorly controlled glycemia introduced any bias into our findings. However, we adjusted for glucose levels before GET, which could affect GET, in all patients to minimize potential confounding factors.

## Conclusions

Our study offers a comprehensive understanding of the real-world frequency and patterns of DGP. Because there are quite a few cases in actual clinical practice, treatment for DGP should receive attention to improve patients’ outcomes. However, further research with a large sample size and a valid diagnosis tool combined with the detection of symptoms is necessary.

### Supplementary Information

**Additional file 1.**

## Data Availability

All the data generated and/or analyzed during the current study are included in this article and are available from the corresponding author on reasonable request.

## Abbreviations

ALT: Alanine aminotransferase
AST: Aspartate aminotransferase
BMI: Body mass index
Cr: Creatinine
DGP: Diabetic gastroparesis
DM: Diabetes mellitus
EMRs: Electronic medical records
FG: Fasting glucose
GET: Gastric emptying time
eGFR: Estimated glomerular filtration rate
GI: Gastrointestinal
HbA1c: Glycated A1c
ICD-10: International Classification of Diseases, 10th Revision
