# Supplementary figures and images for: Gastroparesis might not be uncommon in patients with diabetes mellitus in a real-world clinical setting: a cohort study
Source: BMC Gastroenterol. 2024 Jan 11;24:30. doi: 10.1186/s12876-023-03106-6 (PMC10782575; doi:10.1186/s12876-023-03106-6)

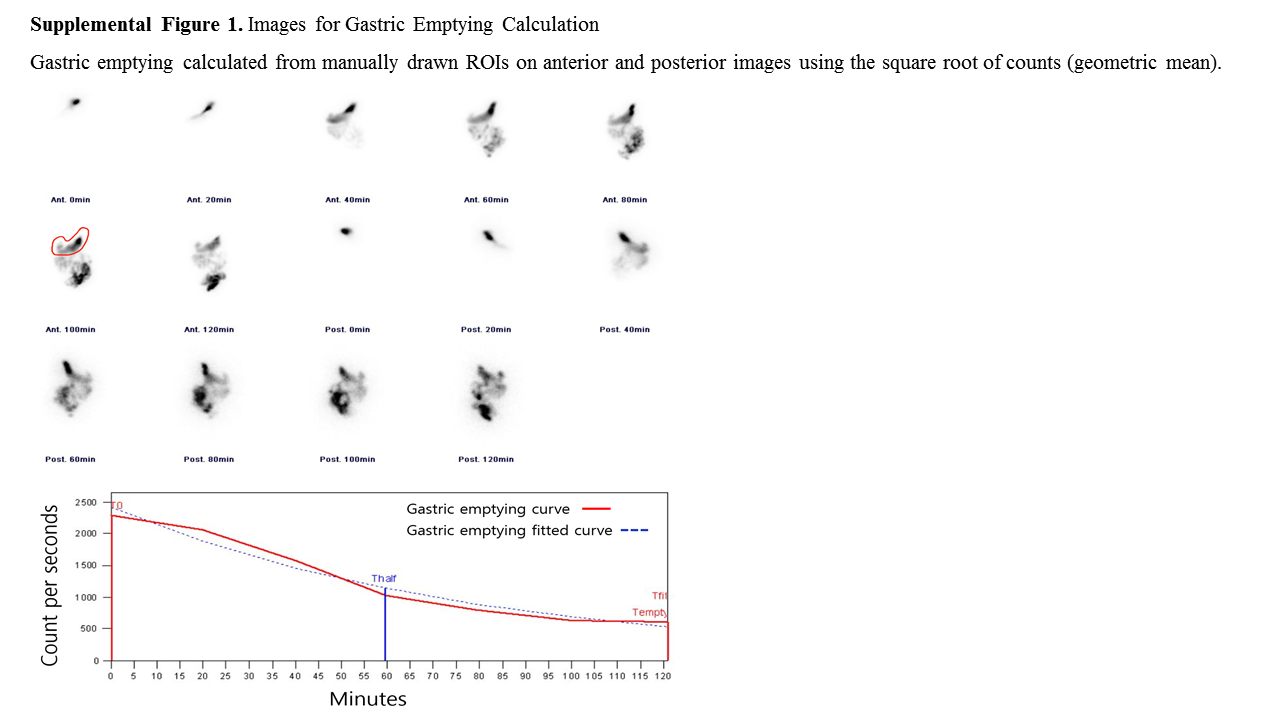

Supplement: Supplementary file 1 — Additional file 1. [file 12876_2023_3106_MOESM1_ESM.tif]
